# Effects of Co on Mechanical Properties and Precipitates in a Novel Secondary−Hardening Steel with Duplex Strengthening of M_2_C and β−NiAl

**DOI:** 10.3390/ma17133261

**Published:** 2024-07-02

**Authors:** Ruming Geng, Shun Han, Xuedong Pang, Xiaoyuan Yuan, Yue Liu, Yong Li, Chunxu Wang

**Affiliations:** 1 Institute for Special Steel Research, Central Iron and Steel Research Institute, Bejing 100081, China; gengruming@nercast.com (R.G.);; 2 Technology Center, Fushun Special Steel Co., Ltd., Fushun 113001, China

**Keywords:** ultra−high strength steel, M_2_C carbide, β−NiAl, atom probe

## Abstract

Synergistic strengthening of nano−scaled M_2_C and β−NiAl has become a new route to develop ultra−high secondary−hardening steel. At present, the effect of Co on the synergistic precipitation behavior of duplex phases of M_2_C and β−NiAl has been rarely reported. This paper revealed the effects of Co on the mechanical properties and duplex precipitates of M_2_C and β−NiAl in a novel 2.5 GPa ultra−high strength secondary−hardening steel. The tensile tests indicated that a 10% Co−alloy steel achieved a much stronger secondary−hardening effects compared to a Co−free steel during aging process, especially in the early−aging state. Needle−shaped M_2_C and spherical β−NiAl particles were observed in both Co−alloy and Co−free steels. However, the number density, and volume fraction of M_2_C were significantly enhanced in the 10% Co−alloy steel. The Mo contents in M_2_C carbide and α−Fe after aging treatment were both analyzed through experimental determination and thermodynamic calculation, and the results indicated that Co decreased the solubility of Mo in α−Fe, thus promoting the precipitation of Mo−rich carbides.

## 1. Introduction

Ultra−high strength steel has been widely used in key load−bearing components of major equipment in the aerospace field, such as aircraft landing gears, rocket engine casings, engine power shafts, transmission shafts, etc. [1,2,3]. At present, ultra−high strength steel mainly includes medium carbon low alloy ultra−high strength steel [4], secondary hardening ultra−high strength steel [5,6], martensitic aging steel [7,8,9], ultra−high strength stainless steel [10], etc. Under the same strength level, secondary−hardening ultra−high strength steel exhibits the best combination of strength, toughness, and fatigue performance.

As a typical representative of secondary−hardening steel, many studies on the microstructures and mechanical properties of AerMet 100 have been reported. There have been many studies on the microstructure and properties of AerMet 100 steel. Through controlling lath martensite with high dislocation density massive nano−scaled M_2_C carbides [11,12,13] and film−shaped reverse austenite [14], the tensile strength of AerMet 100 achieved 1960 MPa, and the fracture toughness reached 115 MPa m^1/2^.

In recent years, utilizing the synergistic strengthening of M_2_C and NiAl to obtain higher strength has also been focused on by many scholars [15,16,17]. Wang [18,19] et al. developed a novel secondary−hardening steel with a tensile strength of 2020 MPa and fracture toughness of 105 MPa·m^1/2^ by controlling the nano−scaled duplex precipitates of M_2_C and β−NiAl. Compared to the AerMet 100, the novel steel obtained an equivalent strength and toughness, while the contents of Ni and Co decreased from 24% to 14%, indicating a significant reduction in alloy cost. Liu [20] et al. also developed a novel 2.4 GPa extra−high strength steel with good ductility of 11.4% total elongation. Zhu [21] et al. also reported a novel cost−effective secondary−hardening steel with an ultimate tensile strength of 2185 MPa, due to the shear strengthening of NiAl and Orowan strengthening of M2C carbides. The excellent mechanical properties of the steel could be well interpreted by synergistic strengthening of high−density dislocations, massive nano−sized M_2_C, and NiAl precipitates.

The precipitation behavior of second phases during the aging process has a significant impact on the mechanical properties of secondary−hardening ultra−high strength steel, while Co has an important impact on the precipiatates. Speich [22] et al. and Heo [23] et al. indicated that Co could inhibit the recovery of dislocations during the aging process, providing more nucleation positions for strengthening precipitates. Won [24] et al. also indicated that Co increased particle number density and volume fraction of M_2_C, thus increasing the degree of secondary hardening. Liu [25] et al. reported that Co enhaced α’−Cr precipitates and accelerated precipitation kinetics of the Mo−rich phase during early−stage aging. Wang [26] et al. indicated that Co addition enhanced the precipitation of fully coherent B2−Ni(Al, Fe) and Mo−rich clusters via depleting both residual Al and Mo in the matrix. Overall, the addition of Co in ferrite decreases the chemical potential of alloying elements due to magnetic interaction and increased the activity of C and Mo elements, thus increasing the driving force of M_2_C carbides, leading to a decrease in the solubility of Mo in ferrite and enhancement of the precipitation of M_2_C carbides.

The synergistic strengthening of carbides and intermetallic compounds has become a new route for further developing higher strength secondary−hardening steel. However, the effect of Co on the synergistic precipitation behavior of duplex phases of M_2_C and β−NiAl was rarely reported.

In the present study, a novel secondary−hardening steel with duplex strengthening of M_2_C and β−NiAl has been designed, obtaining an ultra−high strength of 2.5 GPa and elongation of 10.5%. Combing experimental observation and thermodynamic calculations, the effects of Co on synergistic precipitates of M_2_C and β−NiAl were characterized and analyzed in detail.

## 2. Materials and Methods

### 2.1. Materials

In the present study, a novel secondary−hardening steel with duplex strengthening of M_2_C and β−NiAl precipitations were designed. Its nominal chemical compositions are listed in Table 1. In order to investigate the effect of Co on mechanical properties and precipitation behavior, a similar steel just without Co addition was also prepared. These two experimental steels (10% Co−alloyed and Co−free) were melted using vacuum−induction and casting, following homogenization treatment at 1200 °C and forged into round bar with a diameter of 15 mm. Then, the as forged steel was solution−treated at 1060 °C for 1 h and immediately oil quenched. Then, it was transferred to a cryogenic bath and held at −73 °C for 2 h to promote the transformation of residual austenite to martensite.

**Figure 1:**
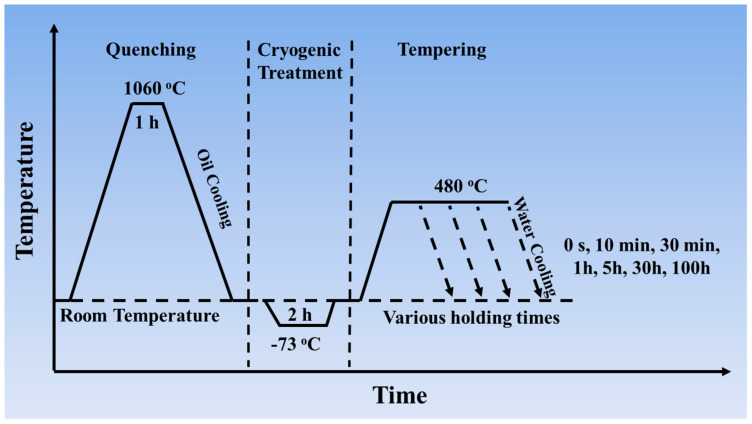
Temperature profiles of the experimental steels during heat treatment.

In order to clarify the mechanical properties of the two steels during the aging process, samples aged at 480 °C for various holding times, including 0 s (after cryogenic treatment), 10 min, 30 min, 1 h, 5 h, 30 h, and 100 h were prepared, and water quenching was chosen after aging treatment. The detailed temperature profiles of heat treatment are shown in Figure 1. Then, the samples were machined into standard specimens for tensile tests and Charpy impact tests.

### 2.2. Experimental Methods

Tensile tests were conducted at room temperature on an MTS−−880 universal material testing machine (MTS−880, MTS Corporation, Woodbury, MN, USA), with a sample size of Φ5 mm × 25 mm and a stretching rate of 1×10^−2^ m/min. Atom probe tomography (APT) tests were performed using a local electrode atom probe (LEAP 4000X Si, CAMECA, Madison, WI, USA ), in which needle−shaped specimens for tests were prepared by a standard two−stage electropolishing procedure. Data reconstruction and analyses were conducted using IVAS 3.8.4. The volume fraction of austenite and dislocation density were measured using an X−ray diffractometer (XRD, Philips APD−−10, Philips Corporation, Amsterdam, The Netherlands). Thin transmission electron microscopy (TEM) foils were prepared from 3 mm diameter discs and ground to 50 μm thickness, then electro−polished. Detailed observation of microstructures and precipitates was conducted using TEM (Hitachi H−−900, Hitachi Corporation, Tokyo, Japan) with an acceleration voltage of 200 kV. Thermodynamic calculations were carried out using Thermo−Calc 2022 software (Thermo−Calc AB Corporation, Stockholm, Sweden) with the TCFE10 database.

## 3. Results and Discussion

**Figure 2:**
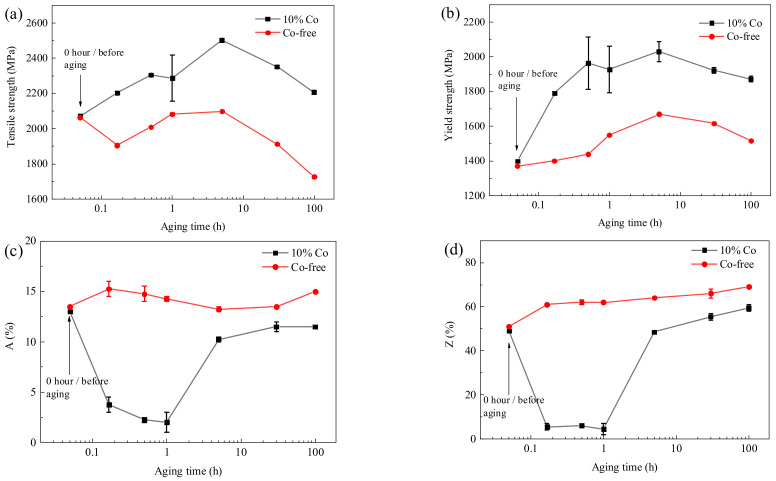
Tensile performance during the aging process. (**a**) Tensile strength, (**b**) yield strength, (**c**) elongation, and (**d**) area reduction.

Figure 2 illustrates the variation in tensile properties during the aging process of the two steels. Initially, both materials exhibited comparable strength and ductility before aging treatment. As depicted in Figure 2a, the 10% Co−alloyed steel exhibited a continual increase in tensile strength with extended aging time, achieving a peak tensile strength of 2508 MPa at 5 h. Subsequently, further increasing the aging time to 30 h and 100 h led to a decrease in tensile strength. However, for the Co−free steel, an obvious drop in tensile strength was noted after 30 min of aging, and then reached its peak strength at 5 h. Through the precipitation strengthening, a strength improvement greater than the cryogenic specimen was obtained during the aging process, thereby contributing to the secondary hardening effect. Notably, the aging peak strength of the Co−free steel was comparable to that of the cryogenic specimen, while Co significantly enhanced the secondary hardening effect. This change of strength in the early aging was consistent with the results of Liu [25] et al. Figure 2b shows the evolution of yield strength. It can be seen that the yield strength gradually increased with the extension of aging time. A significantly higher yield strength was obtained in Co−containing steel.

Figure 2c,d show the elongation and area reduction of the experimental steels. It is evident that the ductility of the Co−free steel generally increased with increasing aging time. In contrast, for the 10% Co steel, when the aging time was less than 1 h, the elongation and reduction of area were less than 5% and 10%, respectively, exhibiting brittle fracture. When the aging time reached 5 h or more, good ductility was achieved. The ductility of the Co−free steel was superior to that of the 10% Co steel. Delagnes [17] et al. and Liu [25] et al. also reported similar results.

Overall, through the duplex strengthening of M_2_C and β−NiAl, a novel secondary−hardening steel with good balance of strength and plasticity has been developed in the present study, with a tensile strength of 2.5 GPa, an elongation of 10.5%, and an area reduction near to 50%.

**Figure 3:**
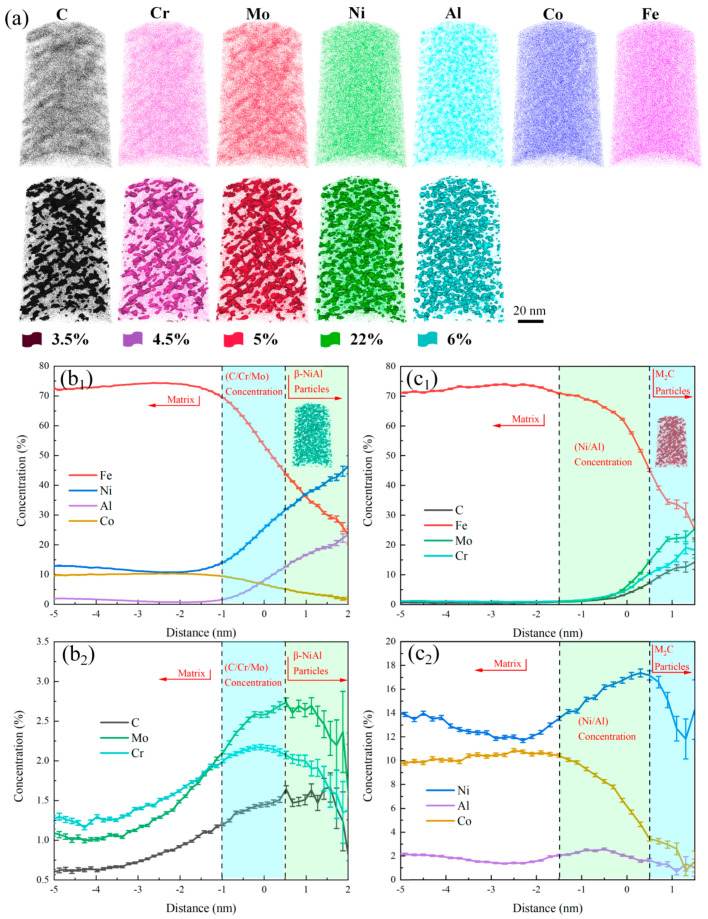
APT results of 10 % Co−alloyed steel after aging for 5 h. (**a**) Three−dimensional spatial distribution maps of elements, flags represent corresponding elements atom concentrations. (**b_1_**,**b_2_**) Element concentrations (at. %) of β−NiAl. (**c_1_**,**c_2_**) Element concentrations (at. %) of M_2_C.

**Figure 4:**
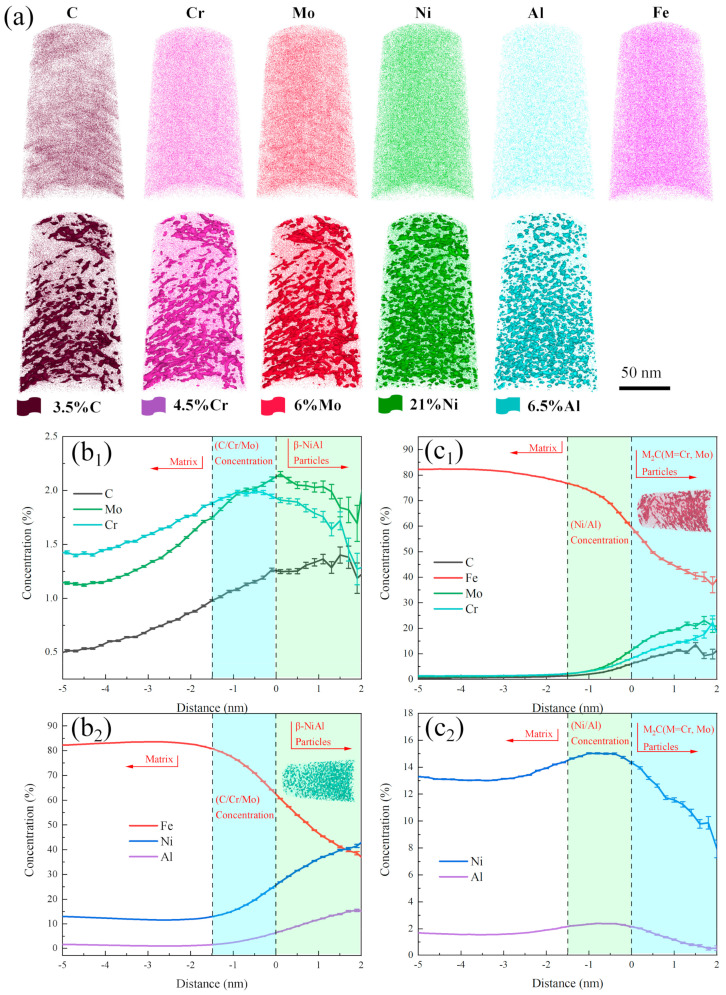
APT results of Co−free steel after aging for 5 h. (**a**) Three−dimensional spatial distribution maps of elements, flags represent corresponding elements atom concentrations. (**b_1_**,**b_2_**) Element concentrations (at. %) of β−NiAl. (**c_1_**,**c_2_**) Element concentrations (at. %) of M_2_C.

Samples of 10% Co−alloyed steel and Co−free steel after aging for 5 h were selected for precipitation phase analysis using 3DAP, and the results are shown in Figure 3 and Figure 4, respectively. Through the elements distribution maps in Figure 3a, it was found that after aging for 5 h, C, Cr, Mo, Ni, and Al elements all exhibited significant segregation. The distribution of C, Cr, and Mo were relatively consistent, as the distribution of Ni and Al were also relatively consistent, indicating the formation of needle−shaped M_2_C and spherical β−NiAl precipitates. And the same phenomenon was also observed in Co−free steel, as shown in Figure 4a.

The composition distribution of individual β−NiAl and M_2_C precipitates are given in (b) and (c) in Figure 3 and Figure 4, respectively. For β−NiAl, Ni and Al contents gradually increased from the edge to the center, while the C, Cr, and Mo contents reached maximum in the transitional region between β−NiAl and the matrix, then decreased in the core of β−NiAl. It may indicate that C, Cr, and Mo are expelled from the core with the nucleation and growth of β−NiAl. It can also be clearly observed that Ni and Al elements were enriched in the transition zone between M_2_C carbide and the matrix.

Based on the characterization results of 3D−APT, the volume fraction, number density, average volume, and nominal radius of precipitates in the two experimental steels were further statistically analyzed, as listed in Table 2. It can be seen that with the addition of 10% Co, the volume fraction of M_2_C significantly increased from 1.09% to 1.99%, and the number density also increased from 2.734 × 10^17^/cm^3^ to 5.596 × 10^17^/cm^3^. The volume fraction of β−NiAl remained basically unchanged and its number density slightly increased. Moreover, the average volumes of individual M_2_C and β−NiAl decreased.

**Figure 5:**
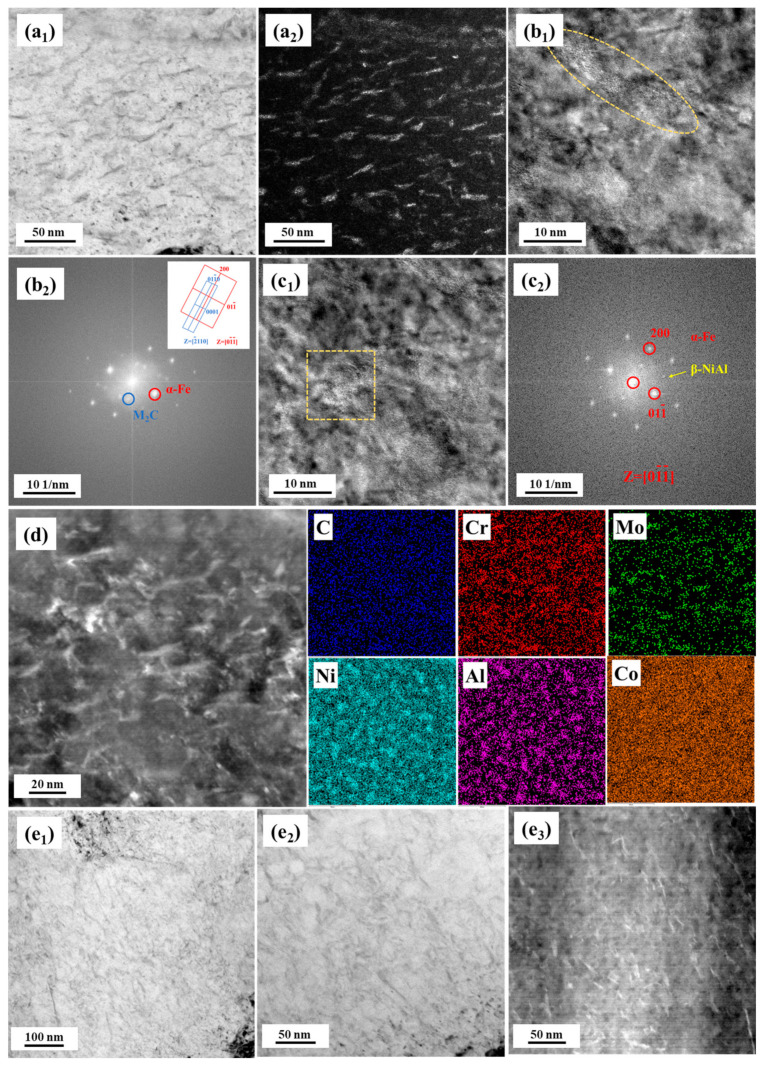
TEM images of precipitates in the steel after aging for 100 h. (**a**–**d**) 10% Co−alloyed steel, (**e**) Co−free steel. (**a_1_**,**a_2_**) bright field and dark field images, (**b_1_**) high−resolution TEM image, (**b_2_**) fast Fourier transform (FFT) of M_2_C corresponding to yellow circle in (**b_1_**), (**c_1_**,) high−resolution TEM image, (**c_2_**) FFT of β−NiAl corresponding to yellow square in (**c_1_**), (**d**) element maps, and (**e_1_**–**e_3_**) bright field and dark field images.

Because of the too small a size of the precipitate after aging for 5 h, samples after aging for 100 h were chosen to further be observed using TEM, as shown in Figure 5. The bright and dark field images indicated that a large number of needle−shaped precipitates existed in the matrix, which could be identified as M_2_C through fast Fourier transform (FFT) and element mappings. For the β−NiAl phase, it was hardly directly observed through bright and dark field images due to a small contrast to the matrix. The FFT image indicated that a series of weak patterns existed at 1/2 of the (200)_α_ direction, which was β−NiAl and exhibited a cube−on−cube relationship with the matrix [27,28]. The obvious element enrichment in Figure 5d also provided evidence of the presence of M_2_C and β−NiAl. From the comparison between Figure 5(a2,e2), it also can be found that the number density of M_2_C in 10% Co−alloyed steel was higher than that in the Co−free steel.

The number density of M_2_C and β−NiAl corresponded to their nucleation number density, which was mainly related to the number of nucleation sites, including dislocations, grain boundaries, vacancies, and pre−existing precipitated particles. Previous study [22,23] has reported that Co can hinder dislocation recovery during the aging process, providing nucleation sites for precipitates. Due to an extremely low mismatch with the α−Fe, the nucleation mode of β−NiAl was mainly heterogeneous nucleation in the matrix [29]. However, the nucleation mode of M_2_C carbide both included nucleation in the matrix and on the β−NiAl particles. Therefore, the formation of β−NiAl can also promote the nucleation of M_2_C carbide, corresponding to a significant increase and a slight increase in number density of M_2_C and β−NiAl, respectively.

**Figure 6:**
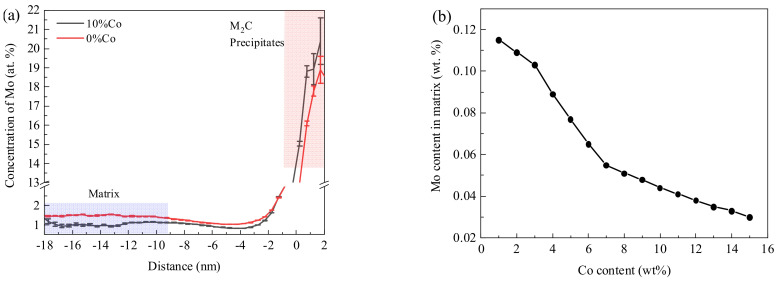
(**a**) Statistical results of Mo contents in matrix and M_2_C carbide through 3D−APT, (**b**) Calculation results of Mo contents in matrix after the aging process with different Co contents using Thermo−Calc software.

The effect of Co on volume fraction of M_2_C carbide was related to the solubility of Mo in the matrix [30]. The Mo contents in M_2_C particles and matrix were analyzed using 3D−APT, and the results are shown in Figure 6a. It can be seen that after adding 10% Co, the Mo content in the matrix significantly decreased as that in the M_2_C carbide increased. The variation of Mo contents in the matrix were also calculated using Thermo−Calc software, and the Co content varied from 1% to 15%, as shown in Figure 6b. It can be seen that as the Co content increased, the Mo content in the matrix gradually decreased, which corresponded with the experimental results.

The above research indicated that the addition of Co can reduce the solubility of Mo in the matrix, promoting much more Mo elements to form precipitates. Moreover, similar conclusions on the effect of Co on the solubility of Mo in maraging steel were also reported by Sha [31] et al.

## 4. Conclusions

The effects of Co on the mechanical properties and duplex precipitates of M_2_C and β−NiAl were investigated in a novel 2.5 GPa ultra−high strength secondary−hardening steel. The 10% Co−alloy steel after quenching and cryogenic treatment obtained a similar strength compared to the Co−free steel, but the peak strength of the former during the aging process was much higher than that of the latter, exhibiting much stronger secondary hardening effects with Co addition. The precipitates characteristics indicated that needle−shaped M_2_C and spherical β−NiAl were formed in both steels. However, the addition of Co significantly increased the number density and volume fraction of M_2_C, and slightly increased the number density of β−NiAl. Experimental determination and thermodynamic calculations indicated that Co can decrease the solubility of Mo in α−Fe, thus promoting the precipitation of Mo−rich carbides.

## Figures and Tables

**Table 1:** Nominal chemical compositions of experimental steel (mass %).

C	Cr	Mo	Ni	Co	Al	Fe
0.28	1.5	2.5	14	10	1	Bal.

**Table 2:** Statistical analysis of precipitates in the two experimental steels.

Precipitates		Co Contents (wt. %)	
		0%Co	10%Co
M_2_C	Volume fraction (%)	1.09	1.99
	Number density (/cm^3^)	2.734 × 10^17^	5.596 × 10^17^
	Average volume (nm^3^)	40.09	35.80
	Nominal radius (nm)	2.12	2.04
β−NiAl	Volume fraction (%)	2.55%	2.49%
	Number density (/cm^3^)	1.085 × 10^18^	1.299 × 10^18^
	Average volume (nm^3^)	23.49	19.17
	Nominal radius (nm)	1.78	1.66

## Data Availability

The original contributions presented in the study are included in the article, further inquiries can be directed to the corresponding authors.

## References

[1] Zhang Y.P., Zhan D.P., Qi X.W., Jiang Z.H. (2018). Austenite and precipitation in secondary−hardening ultra−high−strength stainless steel. Mater. Charact..

[2] Hoshino M., Saitoh N., Muraoka H., Saeki O. (2004). Development of super−9% Ni steel plates with superior low−temperature toughness for LNG storage tanks. Nippon Steel Tech. Rep..

[3] Li J.H., Zhan D.P., Jiang Z.H., Zhang H.S., Yang Y.K., Zhang Y.P. (2023). Progress on improving strength−toughness of ultra−high strength martensitic steels for aerospace applications: A review. J. Mater. Res. Technol. JMRT.

[4] Liu Y.G., Liu J., Li M.Q., Lin H. (2014). The study on kinetics of static recrystallization in the two−stage isothermal compression of 300 M steel. Comput. Mater. Sci..

[5] Wang C.C., Zhang C., Yang Z.G. (2017). Effects of Ni on austenite stability and fracture toughness in high Co−Ni secondary hardening steel. J. Iron Steel Res. Int..

[6] Sun Y.W., Quan J., Salvador H., Lin J., Kozmel T., Mathaudhu S. (2022). Ausforming and tempering of a novel ultra−high strength steel. Mater. Sci. Eng. A.

[7] Niu M.C., Zhou G., Wang W., Shahzad B.M., Shan Y.Y., Yang K. (2019). Precipitate evolution and strengthening behavior during aging process in a 2.5 GPa grade maraging steel. Acta Mater..

[8] Zhang H.L., Ji X., Ma D.P., Tong M., Wang T.J., Xu B., Sun M.Y., Li D.Z. (2021). Effect of aging temperature on the austenite reversion and mechanical properties of a Fe−10Cr−10Ni cryogenic maraging steel. J. Mater. Res. Technol−JMRT.

[9] Han S., Li X.Y., Liu Y., Geng R.M., Lei S.M., Li Y., Wang C.X. (2023). Effect of aging treatment on the microstructure and properties of 2.2 GPa t ungsten−containing maraging steel. Materials.

[10] Zhang Y.P., Zhan D.P., Qi Y.W., Jiang Z.H. (2019). Effect of tempering temperature on the microstructure and properties of ultrahigh strength stainless steel. J. Mater. Sci. Technol..

[11] Manigandan K., Srivatsan T.S., Tammnana D., Poorganji B., Vasudevan V.K. (2024). Influence of microstructure on stain−controlled fatigue and fracture behavior of ultra high strength alloy steel AerMet 100. Mater. Sci. Eng. A.

[12] Raghavan A., Machmeier P. (1998). On the characteristics of M_2_C carbides in the peak hardening regime of AerMet 100 steel. Metall. Mater. Trans. A.

[13] Wen X.R., Zhang J.W., Sun S.H., Guo H., Guo Q.Y., Ding R. (2024). Aging response of a Fe−Cr−Co−Ni secondary hardening steel: The significance of matrix ordering. Mater. Sci. Eng. A.

[14] Shi X.H., Zeng W.D., Zhao Q.Y., Peng W.W., Kang C. (2016). Study on the microstructure and mechanical properties of Aermet 100 steel at the tempering temperature around 482 °C. J. Alloy. Compd..

[15] Garrison W.M., Rhoads M.A. (1996). An evaluation of ultra−high strength steel strengthed by alloy carbide and intermetallic precipitates. Trans. Indian Inst. Met..

[16] Hamano R. (1993). The effect of precipitation of coherent and incoherent precipitates on the ductility and toughness of high −strength steel. Metall. Trans. A.

[17] Delagnes D., Pettinari−Sturmel F., Mathon M.H., Danoix R., Bellot C., Lamesle P., Grellier A. (2012). Cementite−free martensitic steels: A new route to develop high strength/high toughness grades by modifying the conventional precipitation sequence during tempering. Acta Mater..

[18] Gao Y.H., Liu S.Z., Hu X.B., Ren Q.Q., Li Y., Dravid V.P., Wang C.X. (2019). A novel low cost 2000 MPa grade ultra−high strength steel with balanced strength and toughness. Mater. Sci. Eng. A.

[19] Wang C.X., Gao Y.H., Li Y., Han S., Liu S.Z., Zhang P.J. (2020). Effects of solid−solution temperature on microstructure and mechanical properties of a novel 2000 MPa grade ultra−high−strength steel. J. Iron Steel Res. Int..

[20] Liu T.Q., Cao Z.X., Wang H., Wu G.L., Jin J.J., Cao W.Q. (2020). A new 2.4 GPa extra−high strength steel with good ductility and high toughness designed by synergistic strengthening of nano−particles and high−density dislocations. Scr. Mater..

[21] Zhu H.F., Xiong Z.P., Mao J.W., Cheng X.W. (2024). Effect of aging temperature on the microstructural evolution and mehcnaical properties in M_2_C and NiAl co−precipitation secondary hardening ultrahigh−strength steel. J. Mater. Res. Techol..

[22] Speich G.R., Dabkowski D.S., Porter L.F. (1973). Strength and toughness of Fe−10Ni alloys containing C, Cr, Mo, and Co. Metall. Trans..

[23] Heo N.H., Na J.G. (1997). Effect of alloying elements on fracture behavior of Fe−18Ni−2Ti−(8Co) alloys. Met. Mater..

[24] Won Y.J., Kwon Y.J., Moon H.K., Park S.K., Kwon H., Cho K.S. (2018). Secondary hardening behavior in ausformed martensitic alloys with different Co content. Physica B−Condensed Matter.

[25] Liu Z.B., Zhang B.N., Sha G., Jin S.B., Yang Z.Y., Liang J.X., Tian Z.L. (2021). Effect of cobalt on precipitation in Fe−Cr−Co−Mo−Ni−C stainless steels. Mater. Lett..

[26] Wang L.H., Jiang S.H., Peng B., Bai B.H., Liu X.C., Li C.R., Liu X.J., Yuan X.X., Zhu H.H., Wu Y. (2023). Ultrastrong steel strengthened by multiple shearable nanostrcutures. J. Mater. Res. Techol..

[27] Eriach S.D., Leitner H., Brichof M., Clemens H., Danoix F., Lemarchand D., Siller I. (2006). Comparion of NiAl precipitation in a medium carbon secondary hardening steel and C−free PH13−8 maraging steel. Mater. Sci. Eng. A.

[28] Jakob S., Colliander M.H., Kawser J., Rashidi S., Ooi S.W., Thuvander M. (2024). Concomitant precipitation of intermetallic β−NiAl and carbides in a precipitation hardened steel. Metall. Mater. Trans. A.

[29] Sun L., Simm T.H., Martin T.L., Mcadam S., Galvin D.R., Perkins K.M., Bagot P.A.J., Moody M.P., Ooi S.W., Hill P. (2018). A novel ultra−high strength marging steel with balanced ductility and creep resistance achieved by nanoscale β−NiAl and laves phase precipitates. Acta Mater..

[30] Garrison W.M. (2006). A comparison of the effects of cobalt, silicon, nickel and aluminum on the tempering response of a medium chromium secondary hardening steel. ISIJ Int..

[31] Sha W., Cerezo A., Smith G.D.W. (1993). Phase chemistry and precipitation reactions in maraging steels: Part Ⅰ. Introducation and study of Co−containing C−300 steel. Metall. Trans. A.

